# Optical Bandgap Definition via a Modified Form of Urbach’s Rule

**DOI:** 10.3390/ma14071639

**Published:** 2021-03-27

**Authors:** Mithun Bhowmick, Haowen Xi, Bruno Ullrich

**Affiliations:** 1 Department of Mathematical and Physical Sciences, Miami University, Middletown, OH 45042, USA; 2 Department of Physics and Astronomy, Bowling Green State University, Bowling Green, OH 43403, USA; haowenxi@hotmail.com; 3 Ullrich Photonics LLC, Manistique, MI 49854, USA; bruno.ullrich@yahoo.com

**Keywords:** Urbach rule, optical bandgap, semiconductors, InAs, InP, GaAs, InSb

## Abstract

We are reporting an esoteric method to determine the optical bandgap of direct gap materials by employing Urbach’s rule. The latter, which describes the slope of the band tail absorption in semiconductors, in its original version, cannot be employed to pinpoint the optical bandgap. Herein, however, we show that a modified form of Urbach’s rule defines the optical bandgap, and therefore, enables the accurate determination of the optical bandgap energy, which turns out to be identical with the threshold energy for the band tail absorption. The model further produces an explicit expression for the absorption coefficient at the optical bandgap energy.

## 1. Introduction

Models to fit the absorption coefficient *α* of semiconductors as a function of the incident photon energy *E* are important for the design of opto-electronic devices, and, therefore, their improvement and deeper understanding attract ongoing research activities [1]. As pointed out in Ref. [1], which gives an excellent updated overview of the field, conventional models exclude thermal effects, and are based on crystalline materials, resulting for direct absorptive transitions in the well-known relationship below [1,2]:

(1) $$\alpha \left(E\right)=A\sqrt{E-E_{\mathrm{gDOS}}}$$

where *A* is a constant, whose order of magnitude is typically around the saturation of *α*, and *E*_gDOS_ is the theoretical optical bandgap based on the three-dimensional density of states (DOS). However, Equation (1) possesses a considerable shortcoming because it does not consider tail states, which cause a red shift of the absorption edge. Therefore, due to the blurred overlap of Equation (1) with the tail state absorption, the fundamental optical bandgap energy *E*_g_ does not match *E*_gDOS_, specifically not at room temperature [3]. In general, tail state absorption exhibits an exponential decay, and is accounted for by Urbach’s rule [1,2,3,4]:

(2) $$\alpha \left(E\right)=Ke^{\frac{\sigma E}{kT}},$$

where *K* is a constant, *kT* is the thermal lattice energy, and *σ* is a dimensionless parameter, which defines the steepness of the absorption coefficient’s decay. Consequently, the absorption process is divided in two sections, whereas Urbach’s rule is independent from Equation (1) and does not contain information about *E*_g_ [1,2]. Hence, the challenge is to find a model, which conjunct both absorption processes. In Ref. [1], it was achieved by a band-fluctuation model, accurately describing both, the DOS absorption and the Urbach tail region. However, the model is fairly complex and requires numerical and data analysis software tools.

Herein, we reveal a straightforward model based on a modified form of Urbach’s rule, which pinpoints *E*_g_. Beyond that, by fitting *α*(*E*) of InP, InAs, InSb, and GaAs, we demonstrate that the modified form of Equation (2) produces a seamlessly merged joint-DOS (JDOS) with Equation (1).

## 2. The Modified Urbach Rule

Historically, interpreted in detail in Ref. [5], firstly done for the transmission of thin-film CdS, the idea for the modified Urbach rule is based on the analogy to quantum mechanics, which produces continuous wave functions by employing the first derivative continuity condition. Applying the latter principle to Equations (1) and (2), we find the change energy *E*_ch_ (also called the cross-over energy *E*_cr_), where the DOS absorption changes into the Urbach tail [5]:

(3) $$E_{\mathrm{ch}}=E_{\mathrm{gDOS}}+\frac{kT}{2\sigma \;}.$$

In much more recent publications, by fitting the highly transparent part of the transmission spectra of ZnO [3] and GaAs [6], we established that *E*_ch_ = *E*_g_, i.e., Equation (3) transforms to:

(4) $$E_{g}=E_{\mathrm{gDOS}}+\frac{kT}{2\sigma \;},$$

and consequently, the modified Urbach rule is expressed by [3,6]:

(5) $$\alpha \left(E\right)=A\sqrt{\frac{kT}{2\sigma }}e^{\frac{\sigma \left[E-E_{g}\right]}{kT}},$$

describing *α*(*E*) for *E* < *E*_g_, and with Equation (4), Equation (1) alters in the formula below:

(6) $$\alpha \left(E\right)=A\sqrt{E-E_{g}+\frac{kT\;}{2\sigma }},$$

which is valid for *E* ≥ *E*_g_. Equation (6) enables the direct calculation of *α*(*E*_g_), i.e.,:

(7) $$\alpha \left(E_{g}\right)=A\sqrt{\frac{kT\;}{2\sigma }}.$$

We point out that for the limit *T* → 0 K, Equation (6) becomes Equation (1) because of *E*_g_ = *E*_gDOS_, and Equation (7) gives α(*E*_g_) → 0 cm^−1^. In other words, as expected, the lower the temperature the closer are the values of *E*_g_ and *E*_gDOS_, while for *T* → 0 K the tail state absorption collapses and only the DOS absorption remains.

As a consequence of Equation (5), we can express *E*_g_ with the parameters *A* and *σ* of the modified Urbach rule:

(8) $$E_{g}=E-\frac{kT}{\sigma }ln\left\{\frac{\alpha \left(E\right)}{A}\sqrt{\frac{2\sigma }{kT}}\right\}.$$

## 3. New Results and Established Usage of the Modified Urbach Rule

**Figure 1:**
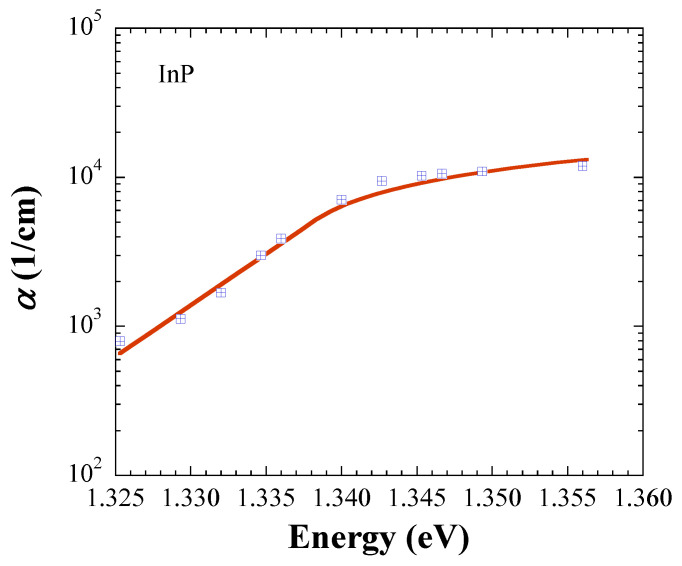
Absorption coefficient of InP. The symbols are retrieved from Ref. [7], and the solid line represents the fit with Equations (5) and (6).

**Figure 2:**
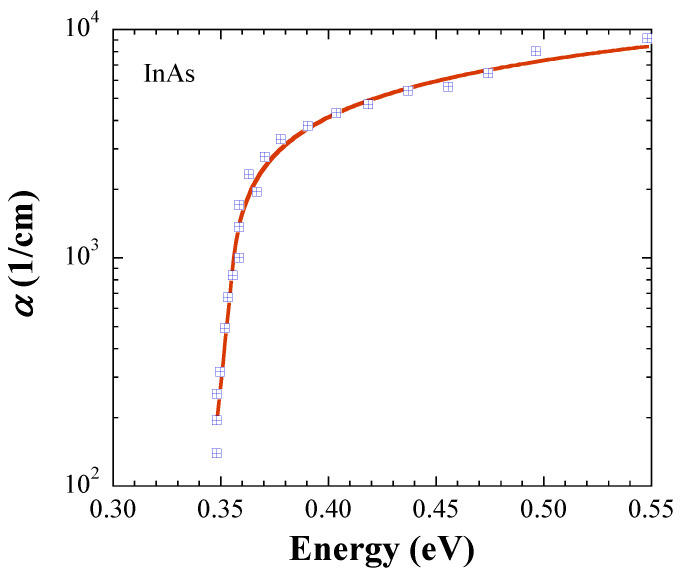
Absorption coefficient of InAs. The symbols are retrieved from Ref. [8], and the solid line represents the fit with Equations (5) and (6).

**Figure 3:**
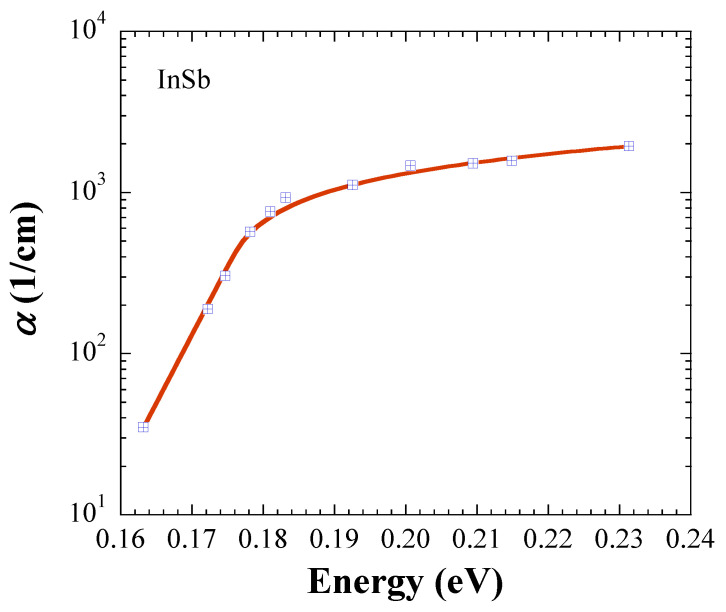
Absorption coefficient of InSb. The symbols are retrieved from Ref. [9], and the solid line represents the fit with Equations (5) and (6).

**Figure 4:**
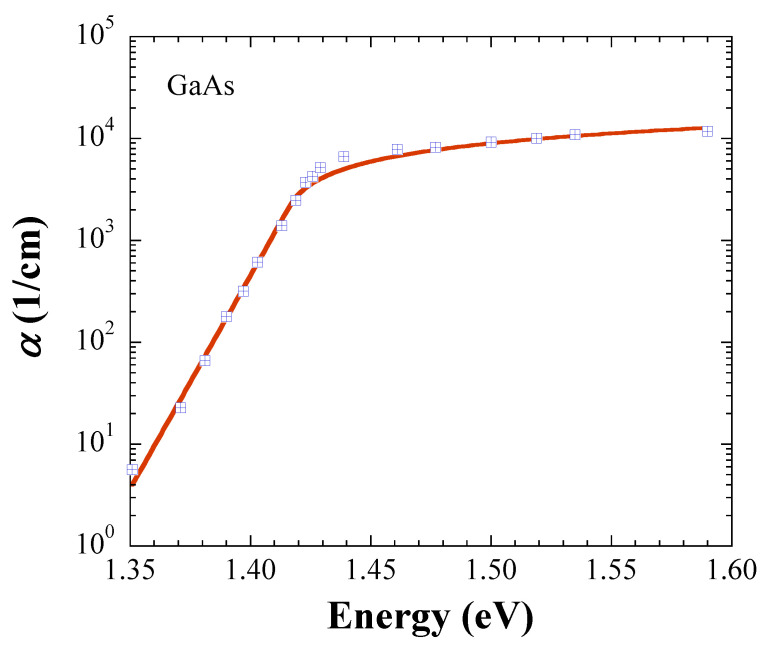
Absorption coefficient of GaAs. The symbols are retrieved from Ref. [10], and the solid line represents the fit with Equations (5) and (6).

In this section, we demonstrate further evidence that the JDOS, which is defined with Equations (5) and (6), results in accurate *E*_g_ values. For this purpose, we retrieved absorption data at room temperature from the literature, and performed fits for crystalline InP [7], InAs [8], InSb [9], and GaAs [10]. The symbols in Figure 1, Figure 2, Figure 3 and Figure 4 represent the measurements, and the solid lines fits with Equations (5) and (6). All fits are carried out with *kT* = 0.025 eV, and Table 1 lists the fit parameters.

For comparison, *E*_g_ values from the literature are listed in Table 2 [11,12]. The fitted *E*_g_ values are in excellent agreement with the literature data for InP, InAs, and InSb, while the fit value of 1.416 ± 0.004 eV for GaAs is 0.014 eV below the numbers given in the literature, but coincides with 1.4287 ± 0.008 eV found by us in Ref. [6]. Table 3 shows *α*(*E*_g_) calculated with the fit parameters in Table 1. Notably, the trend of the In-based semiconductors indicates that *α*(*E*_g_) ∝ *E*_g_, an observation, which justifies further investigation.

## 4. Discussion and Conclusions

While we used the JDOS represented by Equations (5) and (6) for II–VI and III–V compounds quite intensively over the years, among others, in Refs. [3,5,6,13,14,15], the model established popularity within the semiconductor society as well, i.e., other research groups used the model to fit the photoluminescence spectra of CdS nano-ribbons [16] and of InNAs alloys [17], and the absorption and photoconductivity of nanocrystalline bismuth (III) sulfide thin films [18], to refer to some of the appearances in the literature. Additionally, for comparison purposes to the band-fluctuations model in Ref. [1], the JDOS presented herein was used to fit *α(E*) and *E*_g_ of p-type bulk GaAs and nano-crystalline Ga_1−x_Mn_x_N. Notably, referring to Figure 6a,c in Ref. [1], the fits with the band-fluctuation model and Equations (5) and (6) result in practically identical *E*_g_ values for both materials, reinforcing the correctness of our model.

We ought to mention that the only limitation for the presented model, in particular for Equation (1), we encountered when investigating the absorption of ZnS due to the material’s distinguished excitonic absorption, even at room temperature. Notably, however, since excitons do not contribute to photoconducting effects, Equations (5) and (6) describe the absorption transitions forming the photocurrent [15].

Concluding, the work emphasizes that the presented modified form of Urbach’s rule defines *E*_g_, which, according to Equations (3) and (4), is located at the cross-over between the absorption regions reigned by DOS and band tails, respectively. The JDOS presented produces an explicit expression for *α*(*E*_g_), and offers the experimental advantage of strong transmission signals from the highly transmissive spectrum of semiconductors to precisely determine the position of *E*_g_.

## Figures and Tables

**Table 1:** Fit parameter for the fits in Figure 1, Figure 2, Figure 3 and Figure 4.

Bandgap Energy	InP	InAs	InSb	GaAs
*A* (eV^−1/2^cm^−1^)	9 × 10^4^ ± 7560	1.9080 × 10^4^ ± 1488	8 × 10^3^ ± 416	3 × 10^4^ ± 3360
*σ*	3.96 ± 0.36	5.255 ± 0.851	4.885 ± 0.303	2.408 ± 0.130
*E*_gDOS_ (eV)	1.335 ± 0.001	0.353 ± 0.002	0.173 ± 0.001	1.411 ± 0.003
*E*_g_ (eV)	1.338 ± 0.002	0.356 ± 0.003	0.176 ± 0.001	1.416 ± 0.004

**Table 2:** *E*_g_ values retrieved from the literature (first row Ref. [11], and second row Ref. [12]).

Bandgap Energy	InP	InAs	InSb	GaAs
*E*_g_ (eV)	1.34	0.36	0.18	1.43
*E*_g_ (eV)	1.35	0.359	0.17	1.43

**Table 3:** *α*(*E*_g_) calculated with Equation (7) using the fit parameters in Table 1.

-	*α*(*E*_g_) (cm^−1^)
InP	5057 ± 483
InAs	931 ± 105
InSb	405 ± 25
GaAs	2162 ± 249

## Data Availability

The data presented in this study are available on request from the corresponding author.
